# Optimizing COVID-19 Vaccination Strategy in Pediatric Kidney Transplant Recipients: Humoral and Cellular Response to SARS-CoV-2 mRNA Vaccination

**DOI:** 10.3389/ti.2023.11153

**Published:** 2023-05-12

**Authors:** Isabelle Nel, Cyrielle Parmentier, Laurène Dehoux, Marine Minier, Charlotte Duneton, Marina Charbit, Véronique Baudouin, Philippe Bidet, Agnès Carol, Elodie Cheyssac, Jean-Daniel Delbet, Valérie Guérin-El Khourouj, Férielle Louillet, Tim Ulinski, Constance Delaugerre, Guislaine Carcelain, Julien Hogan

**Affiliations:** ^1^ Immunology Department, Robert Debré Hospital, Assistance Publique Hôpitaux de Paris, Paris, France; ^2^ Université Paris Cité, INSERM U976, Paris, France; ^3^ Pediatric Nephrology Department, Armand Trousseau Hospital, Assistance Publique Hôpitaux de Paris, Paris, France; ^4^ Pediatric Nephrology Department, Necker Enfants Malades Hospital, Assistance Publique Hôpitaux de Paris, Paris, France; ^5^ Virology Department, Saint-Louis Hospital, Assistance Publique Hôpitaux de Paris, Paris, France; ^6^ Pediatric Nephrology Department, Robert Debré Hospital, Assistance Publique Hôpitaux de Paris, Paris, France; ^7^ Microbiology Department, Robert Debré Hospital, Assistance Publique Hôpitaux de Paris, Paris, France; ^8^ Pediatric Nephrology Department, Charles Nicolle Hospital, Rouen, France; ^9^ Université Paris Cité, Paris Translational Research Center for Organ Transplantation, INSERM, UMR-S970, Paris, France

**Keywords:** COVID-19, kidney transplantation, children, pediatric, immunology

## Abstract

In this retrospective cohort study, we analyze the early humoral and cellular response in 64 adolescents KTx recipients, after two or three doses of mRNA vaccine BNT162b2 against different variants of COVID-19. After 2 doses, 77.8% % of children with no history of infection had a positive humoral response with a median anti-S IgG level of 1107 (IQR, 593–2,658) BAU/mL. All the patients with a history of infection responded with a higher median IgG level (3,265 (IQR, 1,492–8,178) BAU/mL). In non-responders after 2 doses, 75% responded after a third dose with a median Ab titer at 355 (IQR, 140–3,865 BAU/mL). Neutralizing activity was significantly lower against the delta and the omicron variants compared to the wild-type strain and did not improve after a 3rd dose, while infection did provide higher levels of neutralizations against the variants. T cell specific response correlated with humoral response and no patient displayed a cellular response without a humoral response. Adolescent KTx recipients exhibit a high seroconversion rate after only two doses. A third injection, induces a response in the majority of the non-responders patients but did not counterbalance the strong decrease in neutralizing antibody activities against variants highlighting the need for boosters with specific vaccines.

## Graphical Abstract

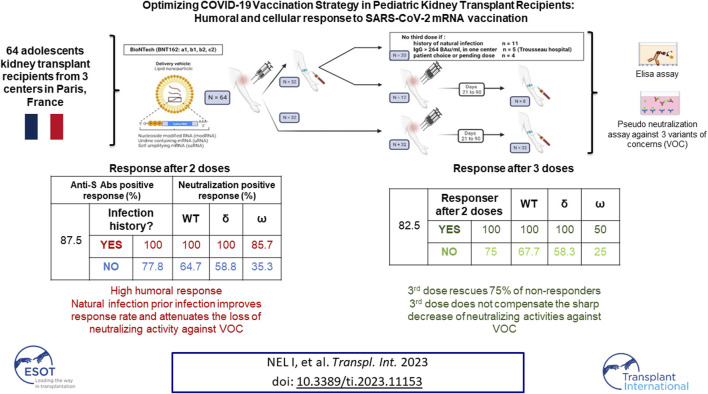

## Introduction

Solid organ transplant (SOT) recipients are at risk of severe complication associated with SARS-COV2 infection [1, 2] and vaccination campaigns in many countries prioritized SOT recipients to receive vaccination. Although, the risk of severe SARS-COV2 infection in pediatric SOT recipients is much lower than in their adult counterparts [3–5] providing pediatric SOT with adequate immunization against SARS-COV2 remains essential.

Previous reports demonstrated poor immunogenicity of mRNA vaccines in adult SOT recipients and especially kidney transplant (kTx) recipients with around 50% of the patients developing anti-spike IgG after two injections [6]. Antibody response improved after a third dose with 60%–70% of the recipients developing anti-spike IgG [7, 8]. This prompted health authorities, in some countries, including France to recommend a third dose of vaccine in adult SOT recipients. T-cell response specific to SARS-COV2 was also studied in adults with conflicting results [7, 9].

The results from a phase 3 safety, immunogenicity, and efficacy data for the Pfizer-BioNTech BNT162b2 mRNA COVID-19 vaccine in healthy adolescents were published in May 2021 [10]. In this study including 2,260 participants aged 12–15 years, antibody titers measured after a 2-dose series met non-inferiority criteria compared with 16- to 25-year-old participant and the tolerance of the vaccine was good. Moreover, full vaccination with 2 doses of Pfizer-BioNTech vaccine was associated with a high efficacy of over 90% in healthy adolescents [11]. This led to the approval of this vaccine for children aged 12–15 in the United States and Europe in May 2021. Data on the immunogenicity of mRNA COVID-19 vaccine in pediatric kTx recipients are scarce and divergent. Sattler et al. reported data on 20 pediatric kTx recipients and found positive antibody titers in 90% of the patients after two doses of BNT162b2 mRNA COVID-19, with 75% developing neutralizing titers against vaccine variant [12]. Another report in older adolescent with kTx reported only 52% of anti-spike IgG after two injections, similar to the results in the adult population [13]. Moreover, there are currently few data on the response to a third dose of mRNA COVID-19 vaccine in pediatric SOT recipients or on SARS-COV2 T-cell specific response following vaccination. These data, but also the neutralizing antibody response against VOC, are needed to assess the optimal vaccination strategy in this population. In this study, we report the immunogenicity of BNT162b2 mRNA by studying humoral response and specific T cells following two or three injections of PfizerBioNTech BNT162b2 mRNA COVID-19 vaccine in pediatric kTx recipients.

## Material and Methods

### Patients

**FIGURE 1:**
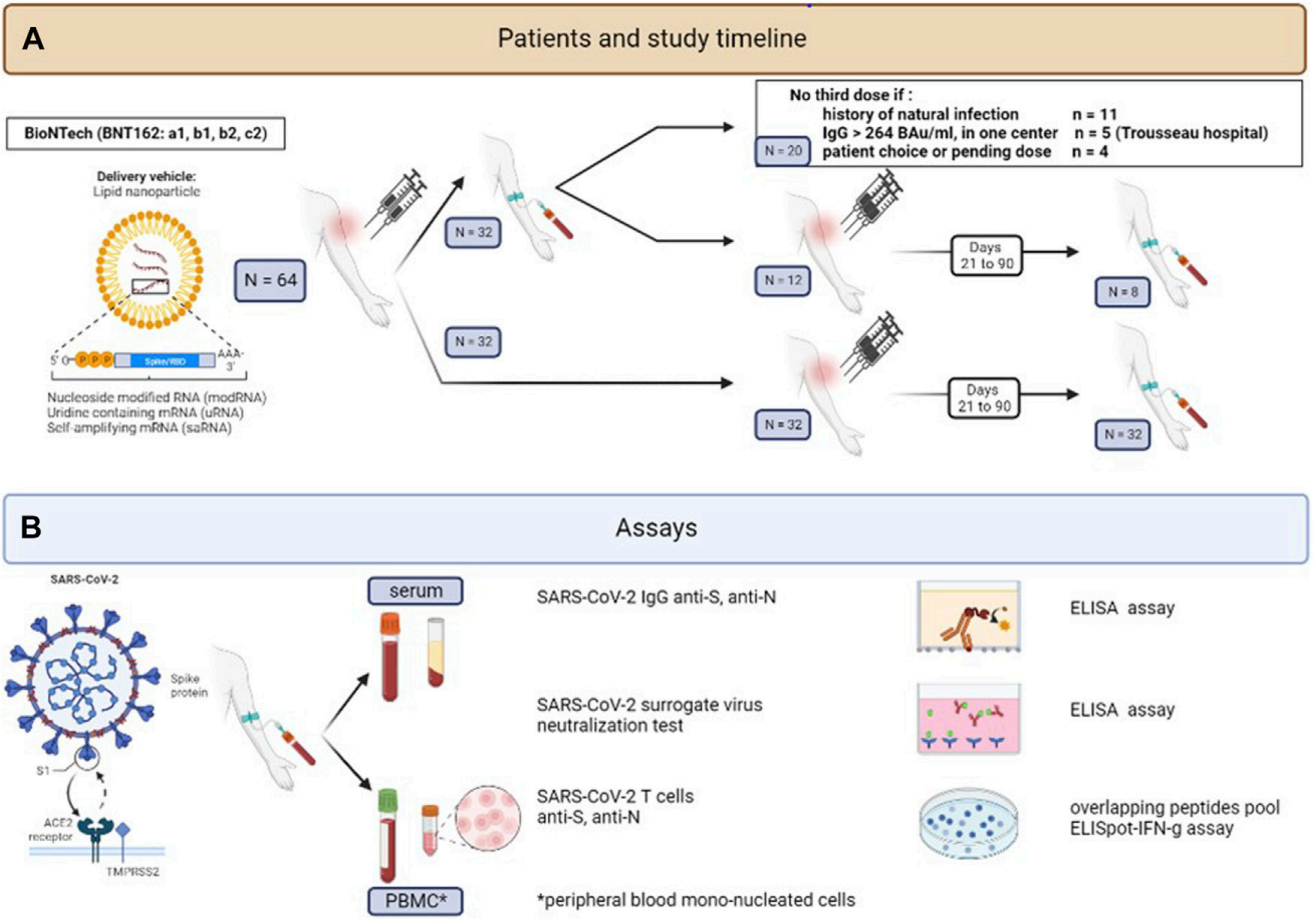
Study design: Study population flow-chart **(A)** and immunological assays **(B)**.

We included all kTx recipients aged over 12 years old followed in one of the three Pediatric Nephrology Departments in Paris (Robert Debré Hospital, Necker Hospital and Trousseau Hospital) who were vaccinated against SARS-CoV-2 with the Pfizer SARS-CoV-2 mRNA BNT162b2 vaccine between 30 January 2021 and 21 December 2021. French health authorities approved vaccination in children with comorbidities more than 16 years old on 20 January 2021 and extended it to children aged 12–15 years old on 01 June 2021. Specific guidelines in adult patients with SOT recommended three injections of mRNA vaccine but no specific pediatric guidelines were available. Therefore, the vaccination strategy was left to the treating physician’s decision with some performing three injections systematically and others only in patients with low anti-S IgG 1 month after the second injection. Patients with a proven (positive SARS-COV2 PCR or home-antigen test) natural infection prior to vaccination only received 2 doses of vaccine (Figure 1A). All centers evaluated patients’ humoral and cellular responses. Blood samples were collected between 21 and 90 days after vaccine injection and processed immediately in a centralized laboratory (Immunology department, Robert Debré Hospital). Clinical and biological data were collected retrospectively. In order to analyze the effect of COVID-19 infection on vaccination, patients were considered as having had an infection if they had a positive PCR, a positive anti-N serology or a positive anti-N T-cell response. The study was approved by Robert Debré Hospital Ethics committee.

### Measurement of Plasma Anti-Spike and Anti-Nucleocapsid SARS-CoV-2 Antibodies

Anti-Spike SARS-CoV-2 antibody levels were evaluated by chemiluminescent immunoassay in plasma using the LIAISON^®^ SARS-CoV-2 TrimericS IgG kit according to manufacturer recommendations (Diasorin^R^). A serological positive response was defined as anti-S IgG response >33.8 BAU/mL. We also present results based on a higher cut-off 264 BAU/mL. This Ab level was found associated with 80% of vaccine efficacy against primary symptomatic COVID-19 (264 BAU/mL) in previous studies [14]. In some patients, serological response after the 2nd vaccine injection was tested in an outside laboratory. These results were collected and used to classify patients in responders and non-responders, but these patients were excluded from the comparison of Ab titers. Anti-Nucleocapsid SARS-CoV-2 IgG levels were evaluated by chemiluminescent immunoassay in plasma using the Alinity I^R^ anti-N SARS-CoV-2 IgG kit according to manufacturer recommendations (Abbott^R^). A serological positive response was defined as anti-N IgG index response >1.4 (Figure 1B).

### Measurement of Neutralizing Antibody Activity Against SARS-CoV-2 Strains

Neutralizing antibodies were quantified using the GenScript^R^ SARS-CoV-2 surrogate Virus Neutralization Test (sVNT). Briefly, the kit detects the ability of antibodies in the plasma of patients to block the interaction between the HRP-conjugated SARS-CoV-2 RBD fragment (HRP-RBD) and the human ACE2 protein (hACE2). Circulating neutralizing antibodies form with HRP-RBD a complex that get removed during washing. Unbound HRP-RBD is captured on a hACE2 pre-coated plate and reacts with the added TMB by changing the color of the solution. The absorbance is inversely dependent on the level of cirulating neutralizing antibodies. HRB-RBD protein used in the kit is selected based on the strain of SARS-CoV-2 tested (wild-type, Delta or Omicron). A positive neutralizing antibody activity is defined as more than 30% according to manufacturer recommendations. Serum from one of the 32 kTx children followed after two vaccine doses was not available for measuring the neutralizing antibody response against SARS-CoV-2 strains (Figure 1B).

### Quantification of Anti-Spike and Anti-Nucleocapsid SARS-CoV-2 T Cells

Human peripheral blood mono-nucleated cells (PBMCs) were isolated from fresh blood samples by density gradient centrifugation (Leucosep) to obtain a final cell concentration of 2.5 10^6^ PBMCs/mL in AIM-V-Medium (Ficher Scientific, Suisse). Anti-Spike and anti-Nucleocapsid cellular responses were evaluated on fresh cells using the T-spot COVID ELIspot system from Oxford Immunotec^R^ (United Kingdom). Briefly, 50 µL of PHA (positive control), AIM-V-Medium (negative control), Spike-antigen mix and Nucleocapsid-antigen mix were added in wells of anti-IFNγ Abs pre-coated plate. 100 μL of the diluted cell suspension were added in each well and the plate was incubated for 16–20 h at 37°C, 5% CO_2_. After discarding supernatant, wells were washed three times with PBS. Afterwards, 50 µL of conjugate reagent (anti-IFNγ Abs, conjugated to alkaline phosphatase) were added to each well and incubated for 1 hour at 4°C in the dark. After washing, 50 µL of substrate solution was added to each well at room temperature for 7 min leading to form insoluble precipitate at the site of reaction. Then, the plate was washed and dried. T-spots were counted by an Elispot-reader (Bioreader^R^ 6000-E Biosys, Germany) and results expressed as SFC (Spot Forming Cell)/250,000 PBMCs. Results were positive for a specific antigen if negative control was ≤10 spots, positive control ≥20 spots and the Antigen-Mix >4 spots per well (according to manufacturer recommendations) (Figure 1B).

### Statistical Analysis

Patient characteristics and immunological response to vaccine are presented as medians and interquartile ranges for continuous variables, and counts and percent for categorical variables. Chi-square test and Mann-Whitney U test were used to compare characteristics between the groups for categorical and continuous variables, respectively. A significant statistical difference was assumed when the *p*-value was <0.05. All analyses were conducted using GraphPad PRISM version 5.00.288 (GraphPad Software Inc., San Diego, CA, United States) and SAS 9.1. The reporting of the data followed the STROBE statement.

## Results

### Patients’ Characteristics

Table 1 describes patients’ and transplants’ characteristics at the time of the first vaccine injection. 64 patients aged 16.9 years (14.9; 17.6) were included, 49 of whom received their transplant from a deceased donor (76.5%). Patients were 56% male; the first causes of ESKD were urological abnormalities in 31.2%, hereditary nephropathies in 37% and glomerular diseases in 19% of the patients. Fifteen patients were transplanted preemptively and 49 patients were transplanted after a median time on dialysis of 1.56 years (1.11; 2.69) (37 in hemodialysis and 12 in peritoneal dialysis). In maintenance oral immunosuppressive treatment, 36 (56%) patients received an association of tacrolimus with mycophenolate mofetil (MMF) or mycophenolic acid (MPA), 18 (28.1%) tacrolimus with azathioprine and 35 (54.7%) with low doses steroids (median 5 mg) as a third immunosuppressive treatment. At first vaccine, 8 patients had lymphopenia <1,500/mm^3^, 3 had hypogammaglobulinemia < 5 g/L and 26 (40%) had eGFR <60 mL/min.

**TABLE 1:** Patients’ and transplants’ characteristic at the time of the first Pfizer SARS-CoV-2 mRNA BNT162b2 vaccine.

Patients’ characteristics at first vaccine	Patients N = 64
Age (years), median (IQR)	16.9 (14.9; 17.6)
Male, *n* (%)	36 (56)
Primary renal diseases, *n* (%)	
CAKUT	20 (31.2)
Hereditary nephropathy	26 (37)
Glomerulonephritis and immunological diseases	12 (19)
Other	6 (9.3)
Donor type: Deceased donor, *n* (%)	49 (76.5)
Time from transplantation to vaccination (years), median (IQR)	3.8 (1.8; 8.3)
KRT before transplantation, *n* (%)	
Preemptive transplantation	15 (23.4)
Hemodialysis	37 (57.8)
Peritoneal dialysis	12 (18.7)
Induction treatment, *n* (%)	
Anti-thymocyte globulins	13 (20.3)
Anti-CD25	49 (76.5)
Maintenance immunosuppression, *n* (%)	
Tacrolimus	58 (90.5)
MMF/MPA	40 (62.5)
Azathioprine	19 (29.7)
Steroids	35 (54.7)
Steroid dose (Median)	5
mTOR inhibitors	3 (4.7)
Belatacept	1 (1.5)
Known history of natural infection SARS-CoV-2, *n* (%)	9 (14)
Biological data at first vaccine	
Lymphocytes (G/L), median (IQR)	2.3 (1.8; 2.9)
Lymphopenia <1,500/mm^3^, *n* (*%*)	8 (12.5)
IgG (G/L), median (IQR)	9.4 (8.3; 12.3)
eGFR (mL/min/1.73 m^2^, Schwartz 2009 equation), median (IQR)	66 (57; 81)
Tacrolimus trough levels (ng/mL), median (IQR)	5.5 (4.2; 6.8)

All patients were vaccinated with the Pfizer SARS-CoV-2 mRNA BNT162b2 vaccine. Twenty patients received only 2 doses of vaccine including 11 with a known previous natural infection with SARS-CoV-2 (median time from infection to first vaccine injection 132 days IQR [81; 291], and 44 (62.5%) patients without known history of natural infection received three doses (Figure 1B).

### Anti-Spike Antibody Levels in kTx Children After 2 or 3 BNT162b2 Vaccine Doses

**FIGURE 2:**
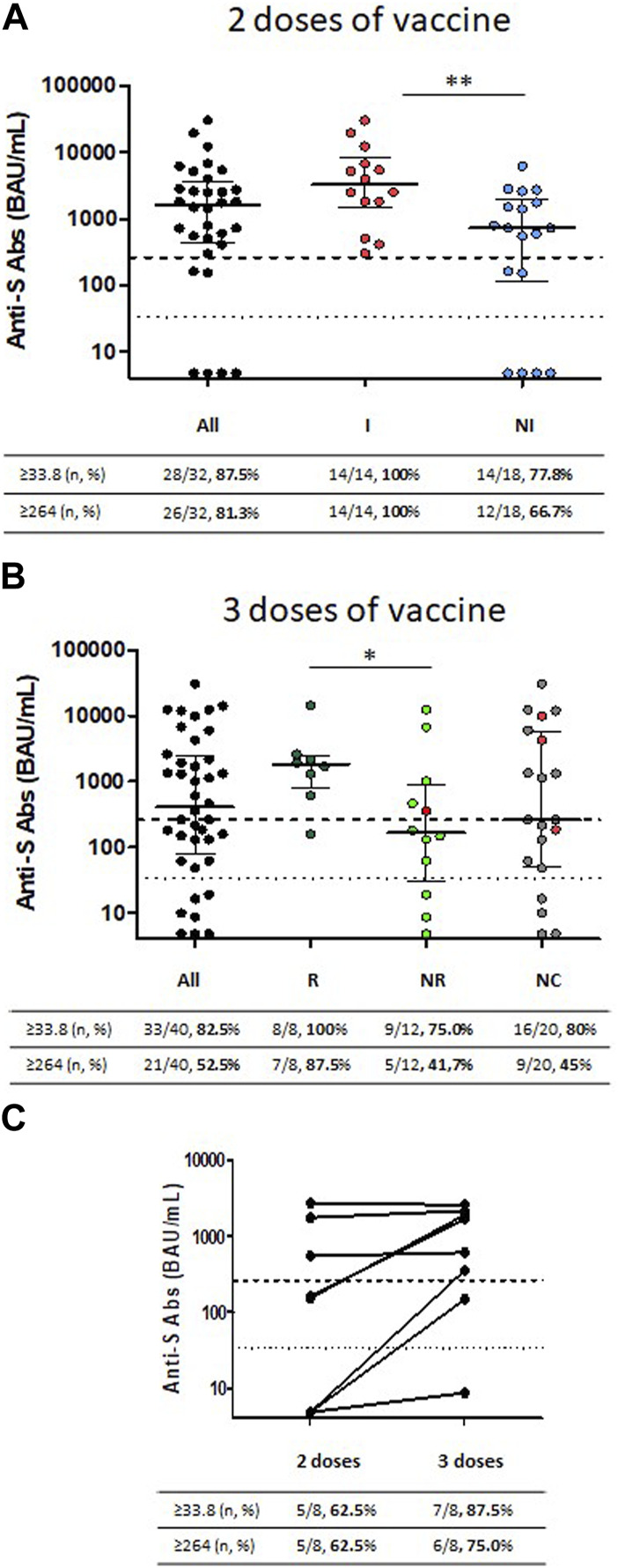
Antibody response to SARS-CoV-2 following two or three injections of SARS-CoV-2 mRNA vaccine in KTx children. **(A)** Titers of anti-S IgG are shown in 32 KTx children following two injections of SARS-CoV-2 mRNA vaccine. Children are classified according to their history of previous natural SARS-CoV-2 infection before the second injection: with natural infection (I) or without natural infection (NI). **(B)** Antibody titers to SARS-CoV-2 following three injections of SARS-CoV-2 mRNA vaccine in 40 KTx children classified according to their humoral response following the second vaccine injection: Responder (R) for positive response, Non-responder (NR) for negative response and Unclassified (NC) for children with missing data. **(C)** Matched SARS-CoV-2 antibody titers for eight patients following the second and third vaccine doses. Dashed horizontal lines represent the threshold of positive humoral response (33.8 BAU/mL) and the ab level associated with 80% of vaccine efficacy against primary symptomatic COVID-19 (264 BAU/mL). Medians and interquartile ranges are shown.

Anti-spike IgG antibodies (anti-S Abs) were quantified in the plasma from 32 kTx children after two doses of vaccine (Figure 2A). 87.5% (28/32) had a positive response (defined as ≥33.8 BAU/mL according to the manufacturer) with a median antibody titer in responders at 1825 (IQR, 637–4,883) BAU/mL. The majority (81.3%, 26/32) had an antibody titer above 264 BAU/mL, Ab levels associated with 80% of vaccine efficacy [14]. KTx children after two vaccine doses were then classified according to their history of natural infection; based either on positive SARS-COV2 PCR or home-antigen test, or on positive anti-N humoral or cellular response. Fourteen of the 32 kTx children evaluated had a history of natural SARS CoV-2 infection before their 2nd vaccine dose and all of them (14/14) had a positive humoral response above the 264 BAU/mL cut-off after vaccination. In comparison, only 77.8% (14/18) of children without previous natural infection had a positive humoral response (*p* = 0.059) and 66.7% (12/18) reaching the 264 BAU/mL cut-off (*p* = 0.017). Among responders, anti-S antibody titers were significantly higher in children with natural infection [median: 3,265 (IQR, 1,492–8,178) BAU/mL] than in children without previous natural infection [median: 1,107 (IQR, 593–2,658) BAU/mL] (*p* = 0.007).

Anti-S Abs were quantified in the plasma for 40 kTx children after three vaccine doses (median time between 3rd dose and serology 39 days IQR (28; 72) (Figure 1A). Serological responses after the second dose of vaccine were determined only for 20 children (for 12 children in a laboratory that provides only qualitative results and for 8 in our central laboratory). Twenty children were only tested after their third dose (Figure 1A). Children were classified in three groups according to their humoral response to the 2nd vaccine dose: Responder for positive response, Non-responder for negative response and Unclassified (NC) for children with missing data. As expected, all (100%, 8/8) responders presented a positive humoral response after their 3rd vaccine dose, with seven (87.5%) achieving Ab levels above the 264 BAU/mL cut-off. Median antibody titer after the 3rd dose in these patients was 1805 (IQR, 783–2,485) BAU/mL. In comparison, the 3rd vaccine dose led to a positive humoral response in 75% (9/12) of non-responders with a lower median Ab titer in responders at 355 (IQR, 140–3,865 BAU/mL) (*p* = 0.028) and only 41.7% of them reaching the 264 BAU/mL cut-off (Figure 2B).

As mentioned above, eight kTx children were tested centrally after the second and the third doses of vaccine including 3 patients with no response after the 2nd dose (Figure 2C). The median antibody titers increased from 159 (IQR, 5–1,458) BAU/mL after 2 vaccine doses to 1,150 (IQR, 201–2,108) BAU/mL after 3 vaccine doses (*p* = 0.085). Ab level above the 264 BAU/mL cut-off were achieved respectively for 5/8 (62.5%) of children after the 2nd dose and 6/8 (75%) children after the 3rd dose. Interestingly, two of the 3 non responders after the 2nd dose developed a positive humoral response after their 3rd vaccine dose, while responders did not show a significant increase in their anti-S IgG titers after the 3rd injection.

### Neutralizing Antibody Response Against Wild-Type, Delta and Omicron SARS-CoV-2 Strains in kTx Children After Two or Three BNT162b2 Vaccine Doses

**FIGURE 3:**
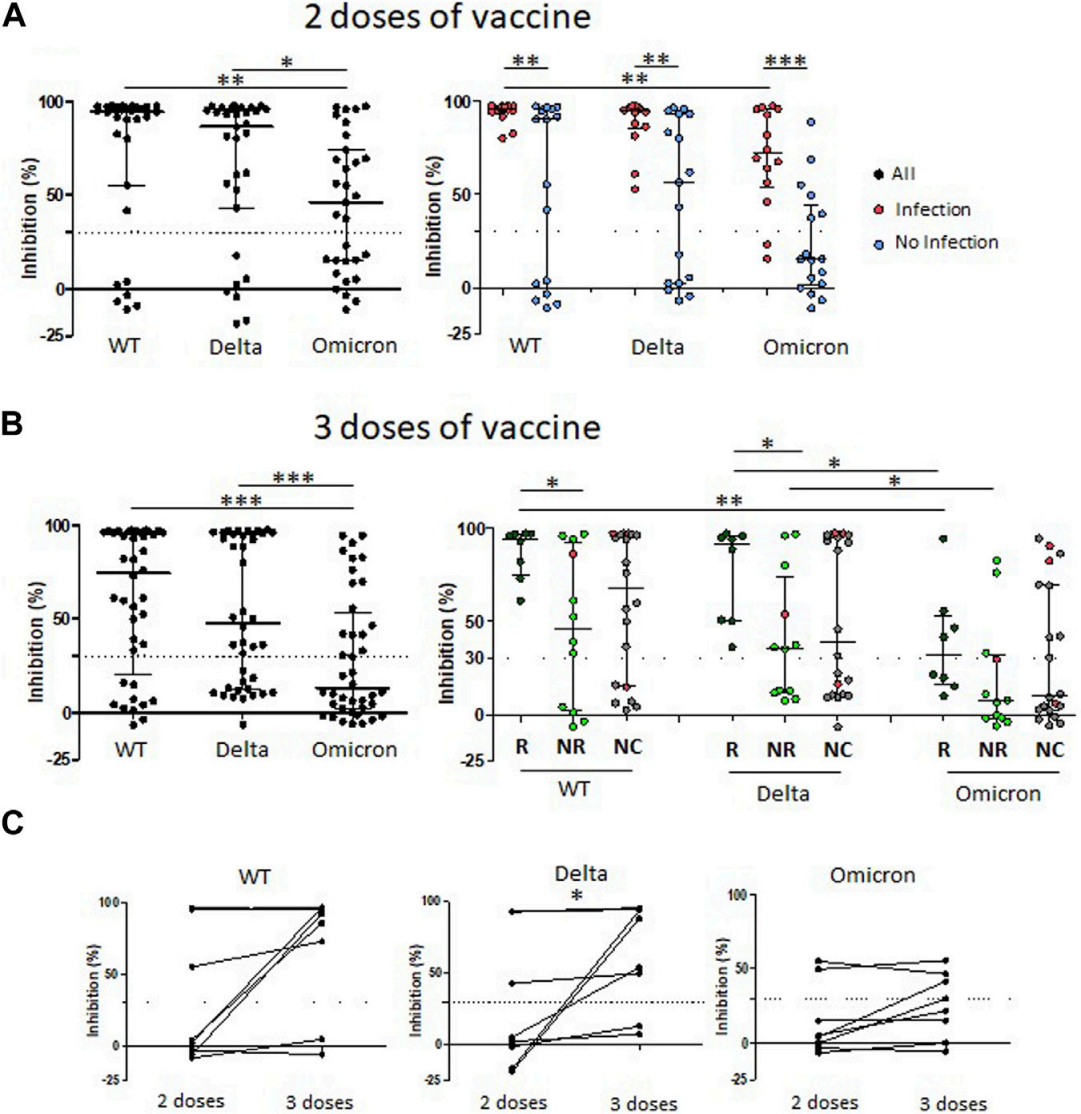
Neutralizing antibody responses against wild-type, Delta and Omicron SARS-CoV-2 strains following two or three injections of SARS-CoV-2 mRNA vaccine in KTx children. **(A)** Neutralizing antibody activities against SARS-CoV-2 strains following two injections of SARS-CoV-2 mRNA vaccine classified according to their previous natural SARS-CoV-2 infection before the second injection: with natural infection (I) or without natural infection (NI). **(B)** Neutralizing antibody activities against SARS-CoV-2 strains following three injections of SARS-CoV-2 mRNA vaccine. Children are classified according to their humoral response following the second vaccine injection: Responder (R) for positive response, Non-responder (NR) for negative response and Unclassified (NC) for children with missing data. **(C)** Matched SARS-CoV-2 antibody neutralizing activities against SARS-CoV-2 strains for eight KTx children following the second and third vaccine doses. Dashed horizontal lines represent the threshold of positive neutralizing antibody response (30%). Medians and interquartile ranges are shown. Anti-SARS-CoV-2 neutralizing antibody activities were evaluated against the wild-type (WT) strain and the Delta and Omicron variants. Medians and interquartile ranges are shown.

Neutralizing antibodies against wild-type, Delta and Omicron strains were quantified using a SARS-CoV-2 pseudo-neutralization Antibody Detection kit (ELISA) [15] in the serum from 31 kTx children after two doses of vaccine (Figure 3A). The positive response (defined as ≥30% according to the manufacturer) against wild-type, Delta and Omicron strains was respectively achieved for 25/31 (80.6%), 24/31 (77.4%) and 18/31 (58.0%) of kTx children. The median neutralizing antibody activity decreased from 95.3% (IQR, 91–96.9) against the wild-type strain to 69.2% (IQR, 53.8–93.5) against Omicron (*p* = 0.003). Among children with a positive neutralizing antibody response, 72% of them had antibodies able to neutralize the three SARS-CoV-2 strains, 24% both the wild-type strain and Delta, and 4% the wild-type strain only (data not shown). All the children with a history of natural SARS-CoV-2 infection before their 2nd vaccine dose (14/14) had a positive neutralizing antibody response against the wild-type strain (Figure 3A, right panel). In comparison, only 64.7% (11/17) of children without previous natural infection had a positive neutralizing response (*p* = 0.008). In agreement, median of neutralizing antibody activity against wild-type strain was significantly stronger in children with natural infection [95.9% (IQR, 93.5–97.3)] than in children without previous natural infection [90.2% (IQR, −0.4–95.9)] (*p* = 0.008). Similar significant differences were observed for neutralizing antibody activity against Delta (*p* = 0.0026) and Omicron strains (*p* = 0.0002). It is however interesting to note that 12/14 (85.7%) of children with natural infection maintained a neutralizing response against Omicron instead of 6/17 (35.3%) of children without previous natural infection.

Neutralizing antibodies against wild-type, Delta and Omicron strains were quantified in the serum for 40 kTx children after three vaccine doses (Figure 3B). 75% (30/40) had a positive response against the wild-type strain. The percentage of children with positive response decreased with variants, reaching 40% (16/40) of children with Omicron (*p* = 0.0015). Similarly, the median neutralizing antibody activity decreased form 93.4% (60.9–96.7) against the wild-type strain to 69.6% (41.8–85.5) against Omicron (*p* = 0.0003). All (100%, 8/8) children with humoral response to the 2nd vaccine dose presented a positive neutralizing response after their 3rd vaccine dose against the wild-type strain with the median of neutralizing activity at 94.5% (IQR, 75.3–96.9) (Figure 3A, right panel). In comparison, the 3rd vaccine dose led to a neutralizing activity in 67.7% (8/12) of children classified non-responders after the 2nd dose, with a lower median at 73.7% (IQR, 42.7–95.4) (*p* = 0.018). Frequency of positive neutralizing response and median neutralizing activity levels decreased from the wild-type strain to Delta, then to Omicron. Only 4/8 (50%) of responders and 3/12 (25%) of non-responders presented a positive neutralizing response against Omicron after their third dose.

As mentioned above, only eight kTx children were centrally evaluated after both the second and the third doses. Among five patients with no neutralizing activity against the wild-type strain after the 2nd dose (Figure 3C), two developed a positive response after their 3rd vaccine dose. The median antibody titers increased from 3.2 (IQR, −5.8–85.8) % after 2 vaccine doses to 89.5 (IQR, 21.6–96.7) %L after 3 vaccine doses (*p* = 0.085). Interestingly, the neutralizing activity against Omicron was not improved after the 3rd injection.

### Correlation Between Anti-Spike Antibody Levels and Neutralizing Antibody Responses Against Wild-Type, Delta and Omicron SARS-CoV-2 Strains in kTx Children After Two or Three BNT162b2 Vaccine Doses

**FIGURE 4:**
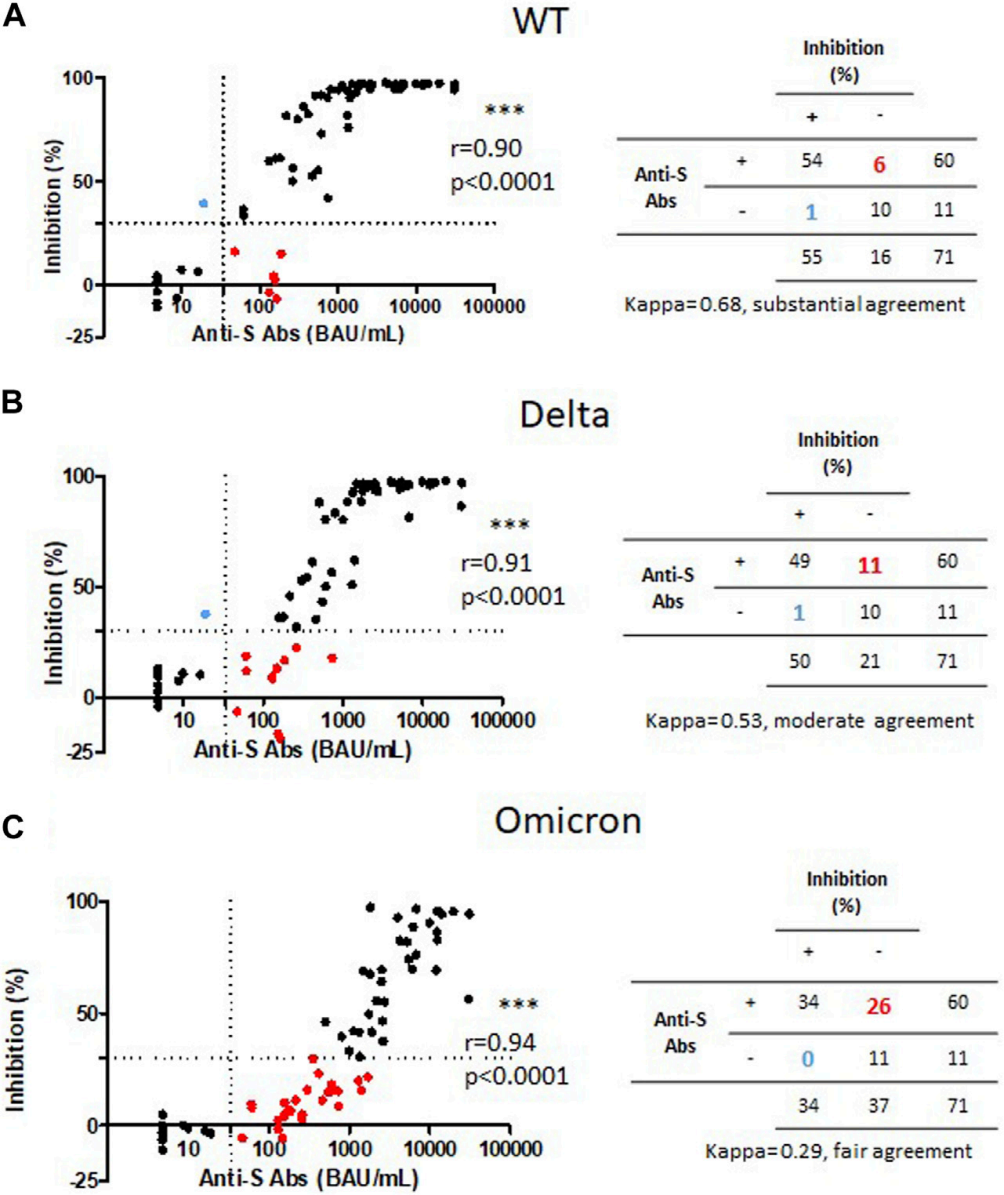
Correlation of anti-S IgG titers and neutralizing antibody activities against the wild-type strain, Delta and Omicron variants of SARS-CoV-2 in KTx children. Correlation of anti-S IgG titers and neutralizing antibody activities against the wild-type strain (WT) **(A)**, Delta **(B)** and Omicron **(C)** strain in 71 KTx children (31 following two injections and 40 following three injections). Dashed lines represent the threshold of positive humoral response (33.8 BAU/mL) and the threshold of positive neutralizing antibody response (30%).

Anti-S antibody levels strongly correlated with neutralizing antibody activity for the wild-type strain (Figure 4A), Delta (Figure 4B) and Omicron (Figure 4C). However, the percentage of children with a positive anti-S antibody response (≥33.8 BAU/mL) without a positive neutralizing activity (cut off 30%) increased from 6/71 for wild-type strain, to 11/71 for Delta, then 27/71 for Omicron. Interestingly, level of anti-S antibody in children without neutralizing activity against the wild type strain were all below the 264 BAU/mL cut-off associated with 80% of vaccine efficacy [14]. Conversely, some patients with high anti-S antibody titers showed no neutralizing activity against the VOCs despite antibody titers up to 736 and 1,690 BAU/mL for delta and omicron variants, respectively.

### Specific Memory T Cells in kTx Children After Two or Three BNT162b2 Vaccine Doses

**FIGURE 5:**
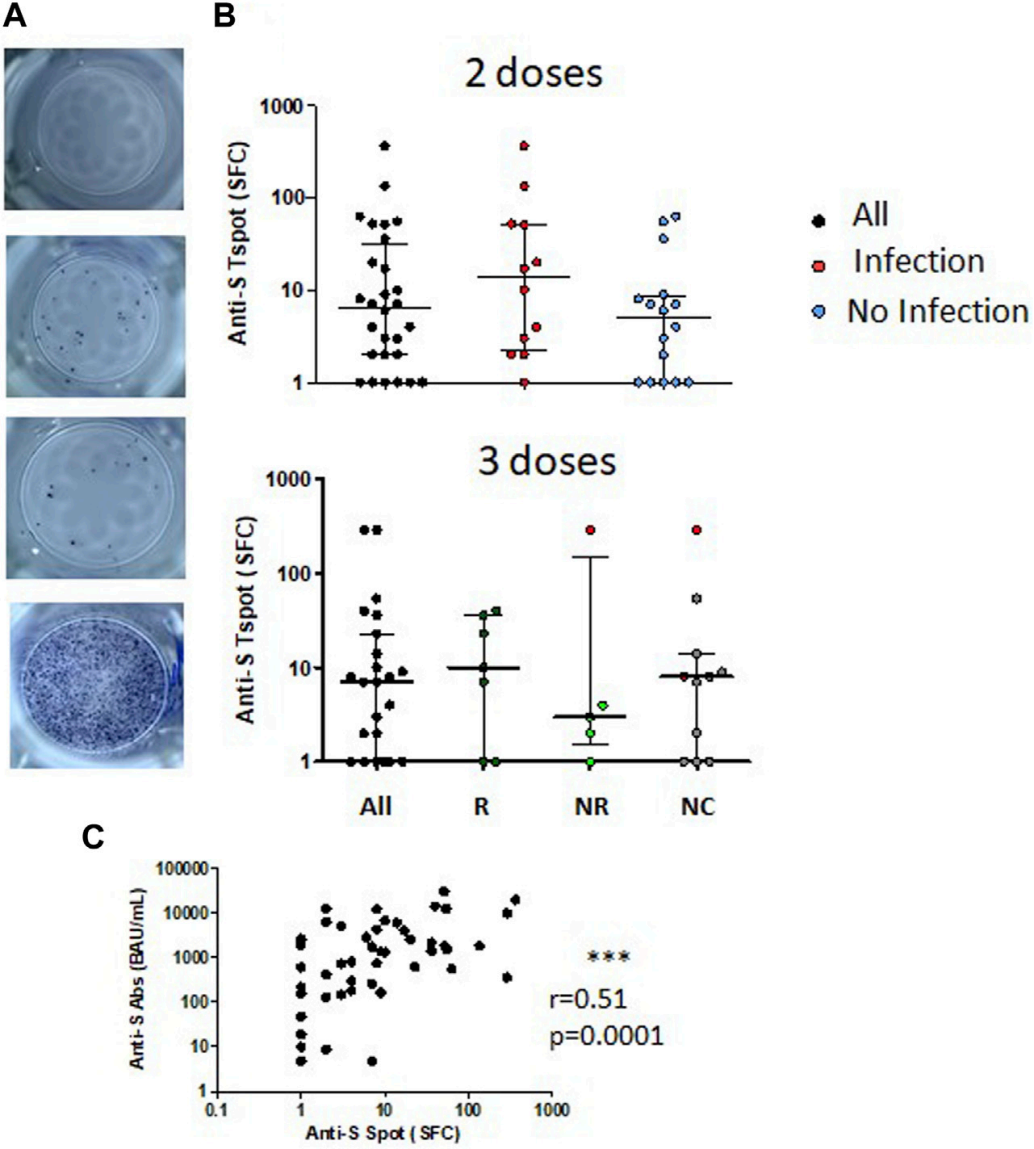
Spike specific T cellular response following two or three injections of SARS-CoV-2 mRNA vaccine in KTx children. **(A)** SARS-CoV-2 specific T cell test results from top to bottom: negative control, anti-S T cell response, anti-N T cell response and positive control. **(B)** Numbers of anti-S T cells are shown in 28 KTx children following two injections of SARS-CoV-2 mRNA vaccine. Children are classified according to their previous natural SARS-CoV-2 infection before the second injection: with natural infection (I) or without natural infection (NI). Numbers of anti-S T cells following three injections of SARS-CoV-2 mRNA vaccine in 23 KTx children classified according to their humoral response following the second vaccine injection: Responder (R) for positive response, Non-responder (NR) for negative response and Unclassified (NC) for children with missing data. Medians and interquartile ranges are shown. **(C)** Correlation of anti-S humoral response and anti-S T cell response in 51 KTx children (28 KTx following two injections and 23 following three injections).

Spike-specific T cells were quantified in 28 kTx children after two vaccine doses and in 23 kTx children after three vaccine doses. Specific spike memory T cell were observed with a median of 6.5 (IQR, 2.0–32) SFC in children after two doses of vaccine. Medians of Spike-specific T cell response were, respectively, 13.5 (IQR, 2.3–51.8) SFC in the 12 children with previous natural infection, and 5 (IQR, 1–8.8) SFC in the 16 children without previous natural infection. Specific memory T cell were observed with a median of 7 (IQR, 1.0–23) SFC in children after three doses of vaccine. Median of Spike-specific T cell response tended to be higher in children with a humoral response after 2 doses (10 (IQR, 1–36) SFC) as compared to non-responders (3 (IQR, 1.5–145.5) SFC). Interestingly, children with the higher level of Spike-specific T cell response in the non-responder group had a natural infection between the 2nd and the third dose (Figures 5A,B).

Among 51 patients tested for both anti-Spike humoral and Spike-specific T cell responses, none had a spike-specific cellular response without a positive humoral response, whereas 9/51 had a positive humoral response without spike-specific cellular response detected (Figure 5C).

## Discussion

In this study, we confirm a higher humoral response rate after two injections of Pfizer SARS-CoV-2 mRNA BNT162b2 vaccine in pediatric kTx recipient (>80%) as compared to the rates reported in adult kTx recipients. We also demonstrate that a third dose of vaccine is able to induce a humoral response in 75% of the children that did not respond after two injections. Moreover, natural infection prior to vaccination significantly improves response rate since all patients with prior infection have stronger humoral responses and neutralizing antibody activities after two injections. Antibodies developed in kTx children with natural infection exhibit a lower loss of neutralizing activity against VOC than in kTx children without infection. Conversely, in responders after two doses, an additional dose of vaccine does not compensate for the sharp decrease in antibody neutralizing activities against VOC. Our data also highlight stronger discrepancies between anti-S IgG levels and neutralizing antibody activities for VOC. Finally, despite immunosuppressive therapy affecting the proliferative and/or effector functions of peripheral T cells, a significant number of kTx children developed anti-S specific T cell response after vaccination.

The high humoral response rate (85%) found in our cohort of pediatric kTx recipients is consistent with the publication of Sattler et al. which report a 90% rate of seroconversion after two doses of BNT162b2 mRNA COVID-19 vaccine among adolescent kTx recipients [16]. These rates are much higher than those reported among adult kTx recipients with around 40% of patients developing anti-spike IgG after two injections [17, 18]. Similarly, Crane et al. and Kermond et al. described lower seroconversion rates, respectively 56% and 50% in adolescent kTx recipients, with a response rate affected by the use of mycophenolate and prednisolone [13, 19]. Individual susceptibility to treatment or differences in treatment regimens may explain the observed discrepancies. As in Sattler et al. report, we observed that approximately 80% of kTx adolescents developed neutralizing antibody against the wild-type strain after two doses of vaccine. Interestingly, we also analyzed the protective responses against variants and showed that neutralizing activity of antibodies decreased with the increasing variability of VOC. Such observation is also described in adult kTx recipients and to a lesser extend in healthy adult donors [18].

The interpretation of low levels of anti-S IgG is already a challenge to predict a clinical protection against the wild-type strain. In our study, the six patients having a positive anti-S IgG response without neutralizing antibody activity, presented anti-S IgG levels below 264 BAU/mL. This cut-off, initially described with the alpha SARS-CoV-2 strain, was found associated with 80% of vaccine efficacy against primary symptomatic COVID-19 in previous studies [14]. Given the major discrepancies between anti-S IgG levels and neutralizing antibody activities against VOC, our results suggest that positivity threshold determined based on data with the alpha strain may not be applicable to other variants. Therefore, the development of assays and/or thresholds specifically designed for new VOC or the assessment of the neutralizing activity against VOC, which will become more accessible with the development of pseudoneutralization test, would be more reliable to evaluate clinical protection against VOC.

We also showed that all patients with a history of COVID-19 infection before vaccination had higher anti-spike antibody titers and neutralizing antibody activities after vaccination than patients without infection. Moreover, they also maintained a better ability to neutralize the Omicron variant compared to patients without infection. Among adult kTx recipients, Magicova et al. reported a major difference in the seroconversion rates between patients with (97%) and patients without (40%) previous infection [20]. Other reports support an improved response to vaccination in infected patients, both in terms of anti-SARS-CoV-2 Ig levels and antibody neutralizing activities, with a better and more sustained clinical protection against new variants [21–23]. Along with our results, this suggests that two injections may be sufficient for the initial vaccination of kTX recipients with a history of COVID-19 infection. However, longitudinal data on the sustainability of the humoral response after various vaccination protocols and after infection are needed to make a definitive conclusion on the best vaccination protocol in these patients.

The analysis of the group of patients receiving three doses demonstrated that a third dose induced a serological response in 75% of the non-responders after two doses. This again contrasts with previous studies in adults reporting much lower rates of seroconversion after a third dose (between 38% [7] and 44% [8]). More importantly, 42% of the non-responders after two doses presented after their third dose of vaccine antibody titers expected to provide an effective protection against severe COVID-19 infections (264 BAU/mL), in line with recent publications suggesting the benefit of a third SARS-CoV-2 vaccine for antibody response in adolescent with kTx [13, 19]. Whether this immunity will last over time and remain effective despite virus variability has to be demonstrated. Indeed, we showed that responders after two doses presented a high neutralizing antibody response against wild-type strain of SARS-CoV-2 but that half of them did not display neutralizing activity against the Omicron stain. Unfortunately, an additional dose of Pfizer SARS-CoV-2 mRNA BNT162b2 vaccine did not overcome the loss of neutralizing activity due to higher virus variability and support the need for new vaccines specific for the variants.

We also assessed T cell specific immunity against SARS-CoV-2. Early in the pandemic, T cell response to mRNA COVID-19 vaccine received a lot of attention. However, only few data on T cell response are available in pediatric kTx recipients. Sattler et al. reported the same frequency of anti-S CD4 T cell in adolescents kTx recipients than in healthy adolescents after vaccination [12]. In our study, despite their immunosuppressive treatment, around 50% of kTx children developed anti-S specific T cell response, after two or three doses of vaccine. Among adult KTx recipients, cell response rates after three doses of vaccine greatly varied from 13% to 85% according to studies [7, 9, 24, 25]. Interestingly, half of kTx children with discrepancies between a positive anti-S humoral response and no neutralizing antibody activities against omicron, developed anti-S specific T cell response (data not shown). The discrepancies between neutralizing activity and T cell response has already been described by Fernandez-Ruiz et al. against the wild-type strain. In their study, the presence of T cell response without any neutralizing antibody activity is described in 13% of patients [26]. The importance of the specific T cell response after COVID-19 vaccination or infection is supported by the demonstration that T cell response is maintained even against variant of concerns (VOCs) [27]. This is indeed of great importance given the impaired neutralizing activity against emerging VOCs in seroconverted kidney transplant recipients after vaccination. This may provide some persistent protection against severe cases of COVID-19 despite substantial loss of neutralizing antibody activity [28].

Altogether, our results show that 1) Pediatric kidney transplant recipients have a high humoral response rate after two injections of Pfizer SARS-CoV-2 mRNA BNT162b2 vaccine, 2) the assessment of the humoral response after two injections is of interest to detect non-responders and perform a third injection, which will induce a response in the majority of the patients, 3) a supplementary vaccine dose did not counterbalance the strong decrease in neutralizing antibody activities against VOC highlighting the need for vaccines against new VOC. Further studies are however needed to assess the impact of the various vaccination strategies and the use of vaccine against new VOC on the maintenance of the immune response.

## Data Availability

The raw data supporting the conclusion of this article will be made available by the authors, without undue reservation.

## Abbreviations

Anti-S Abs, anti-spike antibodies; CAKUT, congenital anomalies of the kidney and the urinary tracts; eGFR, estimated glomerular filtration rate; ESKD, end-stage kidney disease; IgG, immunoglobulin G; kTx, kidney transplant; MMF, mycophenolate mofetil; MPA, mycophenolic acid; PBMC, peripheral blood mono-nucleated cells; SOT, solid organ transplant.

## References

[1] Caillard S Chavarot N Francois H Matignon M Greze C Kamar N Is COVID-19 Infection More Severe in Kidney Transplant Recipients? Am J Transpl (2021) 21(3):1295–303. 10.1111/ajt.16424 PMC7753418 33259686

[2] Kates OS Haydel BM Florman SS Rana MM Chaudhry ZS Ramesh MS Coronavirus Disease 2019 in Solid Organ Transplant: A Multicenter Cohort Study. Clin Infect Dis (2021) 73(11):e4090–e4099. 10.1093/cid/ciaa1097 32766815 PMC7454362

[3] Mastrangelo A Morello W Vidal E Guzzo I Annicchiarico Petruzzelli L Benetti E Impact of COVID-19 Pandemic in Children with CKD or Immunosuppression. Clin J Am Soc Nephrol (2021) 16(3):449–51. 10.2215/CJN.13120820 33318026 PMC8011005

[4] Marlais M Wlodkowski T Vivarelli M Pape L Tönshoff B Schaefer F The Severity of COVID-19 in Children on Immunosuppressive Medication. Lancet Child Adolesc Health (2020) 4(7):e17–8. 10.1016/S2352-4642(20)30145-0 32411815 PMC7220160

[5] Marlais M Wlodkowski T Al-Akash S Ananin P Bandi VK Baudouin V COVID-19 in Children Treated with Immunosuppressive Medication for Kidney Diseases. Arch Dis Child (2020) 106:798–801. Published online December 20. 10.1136/archdischild-2020-320616 33355203 PMC7754669

[6] Boyarsky BJ Werbel WA Avery RK Tobian AAR Massie AB Segev DL Antibody Response to 2-Dose SARS-CoV-2 mRNA Vaccine Series in Solid Organ Transplant Recipients. JAMA (2021) 325(21):2204–6. 10.1001/jama.2021.7489 33950155 PMC8100911

[7] Bertrand D Hamzaoui M Lemée V Lamulle J Laurent C Etienne I Antibody and T-Cell Response to a Third Dose of SARS-CoV-2 mRNA BNT162b2 Vaccine in Kidney Transplant Recipients. Kidney Int (2021) 100(6):1337–40. 10.1016/j.kint.2021.09.014 34619232 PMC8489274

[8] Kamar N Abravanel F Marion O Couat C Izopet J Del Bello A. Three Doses of an mRNA Covid-19 Vaccine in Solid-Organ Transplant Recipients. N Engl J Med (2021) 385(7):661–2. 10.1056/NEJMc2108861 34161700 PMC8262620

[9] Charmetant X Espi M Barba T Ovize A Morelon E Mathieu C Predictive Factors of a Viral Neutralizing Humoral Response after a Third Dose of COVID-19 mRNA Vaccine. *Am J Transpl* Published Online February (2022) 3:1442–50. 10.1111/ajt.16990 PMC10149236 35114060

[10] Frenck RW Klein NP Kitchin N Gurtman A Absalon J Lockhart S Safety, Immunogenicity, and Efficacy of the BNT162b2 Covid-19 Vaccine in Adolescents. N Engl J Med (2021) 385(3):239–50. 10.1056/NEJMoa2107456 34043894 PMC8174030

[11] Olson SM Newhams MM Halasa NB Price AM Boom JA Sahni LC Effectiveness of Pfizer-BioNTech mRNA Vaccination against COVID-19 Hospitalization Among Persons Aged 12-18 Years - United States, June-September 2021. MMWR Morb Mortal Wkly Rep (2021) 70(42):1483–8. 10.15585/mmwr.mm7042e1 34673751 PMC9361838

[12] Sattler A Thumfart J Tóth L Schrezenmeier E Proß V Stahl C SARS-CoV2 mRNA Vaccine-specific B-T- and Humoral Responses in Adolescents after Kidney Transplantation. Transpl Int (2022) 35:10677. 10.3389/ti.2022.10677 35992746 PMC9385879

[13] Crane C Phebus E Ingulli E. Immunologic Response of mRNA SARS-CoV-2 Vaccination in Adolescent Kidney Transplant Recipients. Pediatr Nephrol (2022) 37(2):449–53. 10.1007/s00467-021-05256-9 34522992 PMC8440151

[14] Feng S Phillips DJ White T Sayal H Aley PK Bibi S Correlates of protection against Symptomatic and Asymptomatic SARS-CoV-2 Infection. Nat Med (2021) 27(11):2032–40. 10.1038/s41591-021-01540-1 34588689 PMC8604724

[15] Tan CW Chia WN Qin X Liu P Chen MIC Tiu C A SARS-CoV-2 Surrogate Virus Neutralization Test Based on Antibody-Mediated Blockage of ACE2-Spike Protein-Protein Interaction. Nat Biotechnol (2020) 38(9):1073–8. 10.1038/s41587-020-0631-z 32704169

[16] Qin CX Auerbach SR Charnaya O Danziger-Isakov LA Ebel NH Feldman AG Antibody Response to 2-dose SARS-CoV-2 mRNA Vaccination in Pediatric Solid Organ Transplant Recipients. Am J Transpl (2022) 22(2):669–72. 10.1111/ajt.16841 PMC8653193 34517430

[17] Cirillo L Citera F Mazzierli T Becherucci F Terlizzi V Lodi L Response to Third Dose of Vaccine against SARS-CoV-2 in Adolescent and Young Adult Kidney Transplant Recipients. Transplantation (2022) 106(8):e386–e387. 10.1097/TP.0000000000004199 35581690

[18] Benning L Morath C Bartenschlager M Nusshag C Kälble F Buylaert M Neutralization of SARS-CoV-2 Variants of Concern in Kidney Transplant Recipients after Standard COVID-19 Vaccination. Clin J Am Soc Nephrol (2022) 17(1):98–106. 10.2215/CJN.11820921 34937771 PMC8763153

[19] Kermond RF Ozimek-Kulik JE Kim S Alexander SI Hahn D Kesson A Immunologic Response to SARS-CoV-2 mRNA Vaccination in Pediatric Kidney Transplant Recipients. *Pediatr Nephrol* Published Online July (2022) 14:859–66. 10.1007/s00467-022-05679-y PMC9281214 35833990

[20] Magicova M Zahradka I Fialova M Neskudla T Gurka J Modos I Determinants of Immune Response to Anti-SARS-CoV-2 mRNA Vaccines in Kidney Transplant Recipients: A Prospective Cohort Study. Transplantation (2022) 106(4):842–52. 10.1097/TP.0000000000004044 34999659 PMC8942601

[21] Ali H Alahmad B Al-Shammari AA Alterki A Hammad M Cherian P Previous COVID-19 Infection and Antibody Levels after Vaccination. Front Public Health (2021) 9:778243. 10.3389/fpubh.2021.778243 34926392 PMC8671167

[22] Altarawneh HN Chemaitelly H Ayoub HH Tang P Hasan MR Yassine HM Effects of Previous Infection and Vaccination on Symptomatic Omicron Infections. N Engl J Med (2022) 387(1):21–34. 10.1056/NEJMoa2203965 35704396 PMC9258753

[23] Walls AC Sprouse KR Bowen JE Joshi A Franko N Navarro MJ SARS-CoV-2 Breakthrough Infections Elicit Potent, Broad, and Durable Neutralizing Antibody Responses. Cell (2022) 185(5):872–80. 10.1016/j.cell.2022.01.011 35123650 PMC8769922

[24] Yahav D Rahamimov R Mashraki T Ben-Dor N Steinmetz T Agur T Immune Response to Third Dose BNT162b2 COVID-19 Vaccine Among Kidney Transplant Recipients-A Prospective Study. Transpl Int (2022) 35:10204. 10.3389/ti.2022.10204 35529596 PMC9068869

[25] Devresse A Saad Albichr I Georgery H Yombi JC De Greef J Belkhir L T-Cell and Antibody Response after 2 Doses of the BNT162b2 Vaccine in a Belgian Cohort of Kidney Transplant Recipients. Transplantation (2021) 105(10):e142–e143. 10.1097/TP.0000000000003892 34310103 PMC8487701

[26] Fernández-Ruiz M Almendro-Vázquez P Carretero O Ruiz-Merlo T Laguna-Goya R San Juan R Discordance between SARS-CoV-2–specific Cell-Mediated and Antibody Responses Elicited by mRNA-1273 Vaccine in Kidney and Liver Transplant Recipients. Transplant Direct (2021) 7(12):e794. 10.1097/TXD.0000000000001246 34805496 PMC8601286

[27] Liu J Chandrashekar A Sellers D Barrett J Jacob-Dolan C Lifton M Vaccines Elicit Highly Conserved Cellular Immunity to SARS-CoV-2 Omicron. Nature (2022) 603(7901):493–6. 10.1038/s41586-022-04465-y 35102312 PMC8930761

[28] Riou C Keeton R Moyo-Gwete T Hermanus T Kgagudi P Baguma R Escape from Recognition of SARS-CoV-2 Variant Spike Epitopes but Overall Preservation of T Cell Immunity. Sci Transl Med (2022) 14(631):eabj6824. 10.1126/scitranslmed.abj6824 34931886 PMC9434381

